# Focal adhesion kinase (FAK) activation by estrogens involves GPER in triple-negative breast cancer cells

**DOI:** 10.1186/s13046-019-1056-8

**Published:** 2019-02-06

**Authors:** Damiano Cosimo Rigiracciolo, Maria Francesca Santolla, Rosamaria Lappano, Adele Vivacqua, Francesca Cirillo, Giulia Raffaella Galli, Marianna Talia, Lucia Muglia, Michele Pellegrino, Nijiro Nohata, Maria Teresa Di Martino, Marcello Maggiolini

**Affiliations:** 1 Department of Pharmacy, Health and Nutritional Sciences, University of Calabria, 87036 Rende, Italy; 2 MSD K.K, Tokyo, 102-8667 Japan; 3 Department of Experimental and Clinical Medicine, Magna Graecia University, 88100 Catanzaro, Italy

**Keywords:** TNBC, MDA-MB 231, SUM159, GPER, G-15, FAK, VS-4718, STAT3, STA21

## Abstract

**Background:**

Focal adhesion kinase (FAK) is a cytoplasmatic protein tyrosine kinase that associates with both integrins and growth factor receptors toward the adhesion, migration and invasion of cancer cells. The G-protein coupled estrogen receptor (GPER) has been involved in the stimulatory action of estrogens in breast tumor. In this study, we have investigated the engagement of FAK by GPER signaling in triple negative breast cancer (TNBC) cells.

**Methods:**

Publicly available large-scale database and patient data sets derived from “The Cancer Genome Atlas” (TCGA; www.cbioportal.org) were used to assess FAK expression in TNBC, non-TNBC tumors and normal breast tissues. MDA-MB 231 and SUM159 TNBC cells were used as model system. The levels of phosphorylated FAK, other transduction mediators and target genes were detected by western blotting analysis. Focal adhesion assay was carried out in order to determine the focal adhesion points and the formation of focal adhesions (FAs). Luciferase assays were performed to evaluate the promoters activity of c-FOS, EGR1 and CTGF upon GPER activation. The mRNA expression of the aforementioned genes was measured by real time-PCR. Boyden chamber and wound healing assays were used in order to evaluate cell migration. The statistical analysis was performed by ANOVA.

**Results:**

We first determined by bioinformatic analysis that the mRNA expression levels of the gene encoding FAK, namely PTK2, is higher in TNBC respect to non-TNBC and normal breast tissues. Next, we found that estrogenic GPER signaling triggers Y397 FAK phosphorylation as well as the increase of focal adhesion points (FAs) in TNBC cells. Besides, we ascertained that GPER and FAK activation are involved in the STAT3 nuclear accumulation and gene expression changes. As biological counterpart, we show that FAK inhibition prevents the migration of TNBC cells upon GPER activation.

**Conclusions:**

The present data provide novel insights regarding the action of FAK in TNBC. Moreover, on the basis of our findings estrogenic GPER signaling may be considered among the transduction mechanisms engaging FAK toward breast cancer progression.

**Electronic supplementary material:**

The online version of this article (10.1186/s13046-019-1056-8) contains supplementary material, which is available to authorized users.

## Background

Significant progresses have been reached in the diagnosis and therapy of breast cancer, nevertheless this malignancy still represents the most common leading cause of cancer-related deaths among women worldwide [1]. One of the major challenges for the treatment of breast cancer is its heterogeneous nature, which reflects the different responses to the therapy [2]. Commonly, breast cancer is classified into four major molecular subtypes and each of these has different risk factors for incidence, therapeutic responses, disease progression and preferential organ sites of metastasis [3]. For instance, the triple negative breast cancer (TNBC) exhibits the resistance to different chemotherapies and represents the most aggressive tumor characterized by a low 5-year survival rate (approximately < 30%) [4]. To date, the rate of relapse and the mortality of patients affected by TNBC results at least in part from tumor cell spreading and the consequent development of metastasis [5]. Signals generated from the interaction between cancer cells and the tumor extracellular matrix (ECM) are considered the most common molecular drivers required for cancer cell migration and invasion [6]. In particular, integrin receptors, G-protein coupled receptors, cytokine receptors and tyrosine kinases receptors, sense changes in ECM composition leading to the activation of numerous subcellular biomechanic structures [7, 8]. Among these, the focal adhesion kinase (FAK, also known as PTK2), has been shown to exert a main role in facilitating and promoting the invasiveness of tumor cells [9–11]. Upon activation by integrin-ECM engagment [8] or GPCR agonists [12], FAK can be phosphorylated at the Y397 residue, which allows the formation of a binding site for many SH2 domain containing molecules like Src [13], PI3K [14], Grb7 [15] and PLCγ [16]. In addition to its function as tyrosine kinase, FAK serves as a scaffolding protein triggering the recruitment of diverse molecules to its tyrosine sites [17, 18]. The multifaceted interactions of FAK with various signal transduction mediators may contribute to the FAK-dependent processes involved in cancer development [19]. Indeed, FAK action has been associated to aggressive cancer features as the cell adhesion and spreading [20–22], the enhancement of cell proliferation and survival [23, 24] and the facilitation of invasive cell phenotypes [25–27]. In this context, it is worth mentioning that FAK was shown to be over-expressed in a wide variety of human malignancies, including invasive and metastatic breast tumors [28–30]. Indeed, increased FAK expression and activity has been correlated with different poor prognostic indicators in breast cancer patients [31, 32]. In this regard, it has been observed that the inhibition of FAK may reduce the metastatic potential of breast cancer cells [33–35], indicating FAK as a promising therapeutic target for the treatment of aggressive malignancies [36]. Neverthless, a better understanding on the molecular mechanisms through which FAK activation may contribute to breast cancer progression is still needed.

In recent years, several studies have characterized the role of the G-protein coupled estrogen receptor (GPER, also known as GPR30) in the context of the rapid actions exerted by estrogens [37–39]. Our and other previous investigations have demonstrated that estrogenic GPER signaling mediates stimulatory effects in both breast cancer cells and the tumor microenvironment [40–44]. In this vein, it has been reported that GPER activation triggers different transduction cascades including the epidermal growth factor receptor (EGFR), the mitogen-activated protein kinase (MAPK), the phosphatidylinositol 3-kinase/protein kinase B (PI3K/AKT), intracellular Ca^2+^ mobilization and cyclic AMP (cAMP) production [38]. GPER was also shown to mediate gene expression changes, important biological responses like cell proliferation and migration and it was found negatively correlated with relapse free survival in breast cancer patients [45, 46].

In the framework of the aforementioned findings, in the current study we have focused on the role of GPER in the regulation of FAK signaling by estrogens using the invasive and metastatic TNBC MDA-MB 231 and SUM159 cells as experimental model. Taking advantage of publicly available large-scale genomics and patient data sets as The Cancer Genome Atlas (TCGA), we have found a higher expression of PTK2 gene encoding FAK in TNBC respect to non-TNBC and normal breast tissues. Next, we have observed that estrogens through GPER triggers Y397 FAK phosphorylation, increase FAs and induce gene expression changes. Corroborating these findings, FAK inhibition prevented the migration skill of MDA-MB 231 and SUM159 cells induced by estrogens via GPER. On the basis of the aforementioned results, GPER contributes to the estrogen-activated FAK signaling. Moreover, our data suggest that the GPER-FAK transduction pathway may be considered in more comprehensive targeted therapies in TNBC.

## Methods

### Publicly database and bioinformatics analysis

The clinical significance of PTK2 (FAK coding gene) in TNBC was assessed by microarray data of NCBI Gene Expression Omnibus (GEO) archive (GSE38959) [pubmed: 23254957] and RNA sequencing data in Invasive Breast Cancer Cohort of TCGA project (The Cancer Genome Atlas: https://cancergenome.nih.gov/) [pubmed: 23000897]. The gene expression data of GEO and TCGA were retrieved on August 2nd, 2018 from GEO (https://www.ncbi.nlm.nih.gov/geo/), cBioportal (http://www.cbioportal.org/) or UCSC Xena (https://xena.ucsc.edu/) [pubmed:23550210 and bioRxiv:326470]. The normalized mRNA expression values in the RNA sequencing data were processed and distributed in log_2_ transformed RSEM (RNA-Seq by Expectation Maximization) values (cBioportal) or log_2_ transformed (RSEM+ 1) (UCSC Xena). The Z-scores of PTK2 mRNA expression data and clinical sample information corresponding to breast cancer patients were collected from cBioportal. The status of ER, PR and HER2 IHCs were used for classification of breast cancer subtypes. The PTK2 High group (mRNA Z-score more than 1) and the PTK2 Low group (mRNA Z-score equal or less than 1) were analyzed by Kaplan–Meier survival curves and log-rank statistics.

### Cell cultures

TNBC cell lines MDA-MB 231 were obtained from ATCC (Manassas, VA, USA). TNBC cell lines SUM159 were kindly provided by Dr. W.T. Khaled, University of Cambridge, UK. Cells were used less than 6 months after resuscitation and routinely tested and authenticated according to the ATCC suggestions. MDA-MB 231 cells were maintained in DMEM/F12 (Dulbecco’s modified Eagle’s medium) (Life Technologies, Milan, Italy) with phenol red, supplemented with 5% FBS and 100 μg/ml of penicillin/streptomycin. SUM159 cells were maintained in DMEM/F12 (Dulbecco’s modified Eagle’s medium) (Life Technologies, Milan, Italy) with phenol red, supplemented with 1 μg/ml of insulin, 1 μg/ml of hydrocortisone, 5% FBS and 100 μg/ml of penicillin/streptomycin. MDA-MB 231 and SUM159 cells were grown in a 37 °C incubator with 5% CO_2_. Cells to be processed for immunoblot and RT-PCR assays were switched to medium without serum and phenol red the day before treatments.

### Reagents and drugs

17β-Estradiol (E2) and PI3K inhibitor Wortmannin (WM) were purchased from Sigma-Aldrich (Milan, Italy). G-1 (1-[4-(− 6-bromobenzol [1,3]diodo-5-yl)-3a,4,5,9b-tetrahidro3H5cyclopenta[c]quinolin-8yl]-ethanone) and G-15 (3aS, 4R, 9bR)-4-(6-bromo-1, 3-benzodioxol-5-yl)-3a,4,5,9b-3H-cyclopenta [c] quinolone were obtained from Tocris Bioscience (Space, Milan, Italy). Src kinase inhibitor PP2 was bought from Selleckchem (DBA, Milan, Italy). MEK inhibitor PD98059 (PD) was purchased from Calbiochem (DBA, Milan, Italy). STAT3 transcription factor signaling inhibitor STA21 and Focal Adhesion Kinase selective inhibitor VS-4718 were bought from Santa Cruz Biotechnology (DBA, Milan, Italy). All the aforementioned compounds were dissolved in dimethyl-sulfoxide (DMSO).

### RNA extraction and real-time PCR

Total RNA was extracted from cell cultures using the TRIzol commercial kit (Life Technologies, Milan, Italy) according to the manufacturer’s protocol. RNA was quantified spectrophotometrically and quality was checked by electrophoresis through agarose gels stained with ethidium bromide. Only samples that were not degraded and showed clear 18 S and 28 S bands under UV light were used for RT-PCR. Total cDNA was synthesized from the RNA by reverse transcription using the murine leukemia virus reverse transcriptase (Life Technologies, Milan, Italy), following the protocol provided by the manufacturer. The expression of selected genes was quantified by real-time PCR using Step One ^(^™^)^ sequence detection system (Applied Biosystems Inc., Milan, Italy), following the manufacturer’s instructions. Gene-specific primers were designed using Primer Express version 2.0 software (Applied Biosystems. Inc., Milan, Italy) and are as follows: human c-FOS Fwd: 5’-CGAGCCCTTTGATGACTTCCT-3′ and Rev.: 5’-GGAGCGGGCTGTCTCAGA-3′; human EGR1 Fwd: 5’-GCCTGCGACATCTGTGGAA-3′ and Rev.: 5’-CGCAAGTGGATCTTGGTATGC-3′; human CTGF Fwd: 5’-ACCTGTGGGATGGGCATCT-3′ and Rev.: 5’-CAGGCGGCTCTGCTTCTCTA-3′; 18S Fwd: 5’-GGCGTCCCCCAACTTCTTA-3 and Rev.: 5’-GGGCATCACAGACCTGTTATT-3′. Assays were performed in triplicate and the RNA expression values were normalized using 18S expression and then calculated as fold induction.

### Plasmids, transfections and luciferase assays

The luciferase reporter plasmid for c-fos encoding a 2. 2-kb 5’upstream fragment of human c-fos was a gift from Dr. K. Nose (Hatanodai, Shinagawa-ku, Tokyo). EGR1-luc plasmid, containing the − 600 to + 12 5′- flanking sequence from the human EGR1 gene, was kindly provided by Dr. Safe (Texas A&M University). The CTGF luciferase reporter plasmid p (− 1999/+ 36)-Luc (CTGF-luc), based on the backbone of vector pGL3-basic (Promega) was a gift from Dr. B. Chaqour [47]. The Renilla luciferase expression vector pRL-TK (Promega, Milan, Italy) was used as internal transfection control. MDA-MB 231 TNBC cells (1 × 10^5^) were plated into 24-well dishes with 500 μl/well culture medium containing 5% FBS. Cell medium was replaced on the day of transfection with serum-free medium and transfection was performed using X-tremeGENE 9 DNA Transfection Reagent as recommended by the manufacture (Sigma–Aldrich) and a mixture containing 0.5 μg of each reporter plasmid and 5 ng of pRL-TK. After 6 h, cells were treated with E2 and G1 alone or in combination with GPER antagonist GA15 or STAT3 inhibitor STA21 and incubated for 18 h. Luciferase activity was measured using the Dual Luciferase Kit (Promega, Milan, Italy) according to the manufacturer’s recommendations. Firefly luciferase activity was normalized to the internal transfection control provided by the Renilla luciferase activity. Normalized relative light unit values obtained from cells treated with vehicle (DMSO) were set as 1-fold induction upon which the activity induced by treatments was calculated.

### Western blotting analysis

MDA-MB 231 and SUM159 cells were grown in 10 cm dishes, exposed to the treatments and then lysed as previously described [48]. Equal amounts of whole protein extract were electrophoresed through a reducing SDS/8 and 10% (w/n) polyacrylamide gels, electroblotted onto a nitrocellulose membrane (Amersham Biosciences, GE Healthcare, Milan, Italy), and probed with primary antibodies against Y397-FAK (Cell Signaling Technology, Milan, Italy), FAK (H-1) (Santa Cruz Biotechnology, DBA, Milan, Italy), phosphorylated ERK1/2 (E-4) (Santa Cruz Biotechnology, DBA, Milan, Italy), ERK2 (C-14) (Santa Cruz Biotechnology, DBA, Milan, Italy), p-AKT1/2/3 (Ser 473)-R (Santa Cruz Biotechnology, DBA, Milan, Italy), AKT/1/2/3 (H-136) (Santa Cruz Biotechnology, DBA, Milan, Italy), c-FOS (H-125) (Santa Cruz Biotechnology, DBA, Milan, Italy), EGR1 (C19) (Santa Cruz Biotechnology, DBA, Milan, Italy), CTGF (Origene, DBA, Milan, Italy) and β-actin (C2) (Santa Cruz Biotechnology, DBA, Milan, Italy). Proteins were detected by horseradish peroxidase-linked secondary antibodies (Santa Cruz Biotechnology, DBA, Milan, Italy) and then revealed using the ECL™ Western Blotting Analysis System (GE Healthcare, Milan, Italy).

### Focal adhesion assay

MDA-MB 231 cells cultured on fibronectin-coated 6 well plates were serum deprived and then treated for 30 min with E2 and G1 alone or in combination with G15, as indicated. Then cells were washed three times with PBS, fixed in 4% paraformaldehyde for 15 min, permeabilized with 0.2% Triton X-100, washed three times with PBS and incubated overnight with or without (negative control) a rabbit primary antibody anti p-FAK (Y397) (Cell Signaling Technology, Milan, Italy). After incubation, the wells were extensively washed with PBS and incubated with donkey anti-rabbit IgG-FITC (1:300; purchased from Alexa Fluor, Life Technologies, Milan, Italy) for 1 h at room temperature. Finally, cells were washed with PBS and incubated in PBS buffer containing 4′, 6-diamidino-2-phenylindole dihydrochloride (DAPI), (1:1000), (Sigma-Aldrich, Milan, Italy) 10 min at room temperature for nuclear staining. FAs images were acquired on the Cytation 3 Cell Imaging Multimode Reader (BioTek, Winooski, VT) and analysed using the software Gen5 (BioTek, Winooski, VT).

### STAT3 nuclear immunofluorescence staining

50% confluent MDA-MB 231 cells grown on 6 well plates were serum-deprived and then treated for 1 h with E2 and G1 alone or in the presence of GPER antagonist G-15 or VS-4718 FAK inhibitor, as indicated. Next, cells were fixed in 4% paraformaldehyde for 15 min at room temperature, permeabilized with 0.2% Triton X-100, washed three times with PBS and incubated overnight with or without (negative control) a rabbit primary antibody against STAT3 (Cell Signaling Technology, Milan, Italy). After incubation, the wells were extensively washed with PBS and incubated with donkey anti-rabbit IgG-FITC (1:400; purchased from Alexa Fluor, Life Technologies, Milan, Italy) for 1 h at room temperature. Finally, cells were washed with PBS and incubated in PBS buffer containing 4′, 6-diamidino-2-phenylindole dihydrochloride (DAPI), (1:1000), (Sigma-Aldrich, Milan, Italy) 10 min at room temperature for nuclear staining. Imaging showing nuclear STAT3 accumulation were acquired on the Cytation 3 Cell Imaging Multimode Reader (BioTek, Winooski, VT) and analysed using the software Gen5 (BioTek, Winooski, VT).

### Transwell migration assay

Migration assay was performed in triplicate using boyden chambers (Costar Transwell, 8 mm polycarbonate membrane, Sigma Aldrich, Milan, Italy). Briefly, MDA-MB 231 and SUM159 cells were seeded onto the upper membrane of the chamber at a density of 2,5 × 10^5^ cells/ml. Next, the cells were exposed to the treatment with E2 or G1 used alone or in combination with GPER antagonist G-15, VS4718 FAK inhibitor or STA21 STAT3 inhibitor. 4 h after seeding, the cells on the bottom side of the membrane, were fixed with paraformaldehyde, permeabilized with methanol and finally stained with GIEMSA for 15 min at room temperature. Cell migrated were counted by using Cytation 3 Cell Imaging Multimode Reader (BioTek, Winooski, VT).

### Scratch wound healing assay

MDA-MB 231 cells were allowed to grow in 6 well/plates in regular medium supplemented with 5% FBS until they reached a 70 to 80% confluence. To create a scratch of the cell monolayer, a p200 pipette tip was used. Cells were then washed twice with PBS to remove the detached cells and treated with the various compounds, as indicated. The migration ability of the cells was evaluated after 24 h of treatments.

### Statistical analysis

The statistical analysis was performed using ANOVA followed by Newman–Keuls’ testing to determine differences in means. *p* < 0.05 was considered as statistically significant.

## Results

### Database analysis of the PTK2 gene encoding FAK in TNBC

**Fig. 1:**
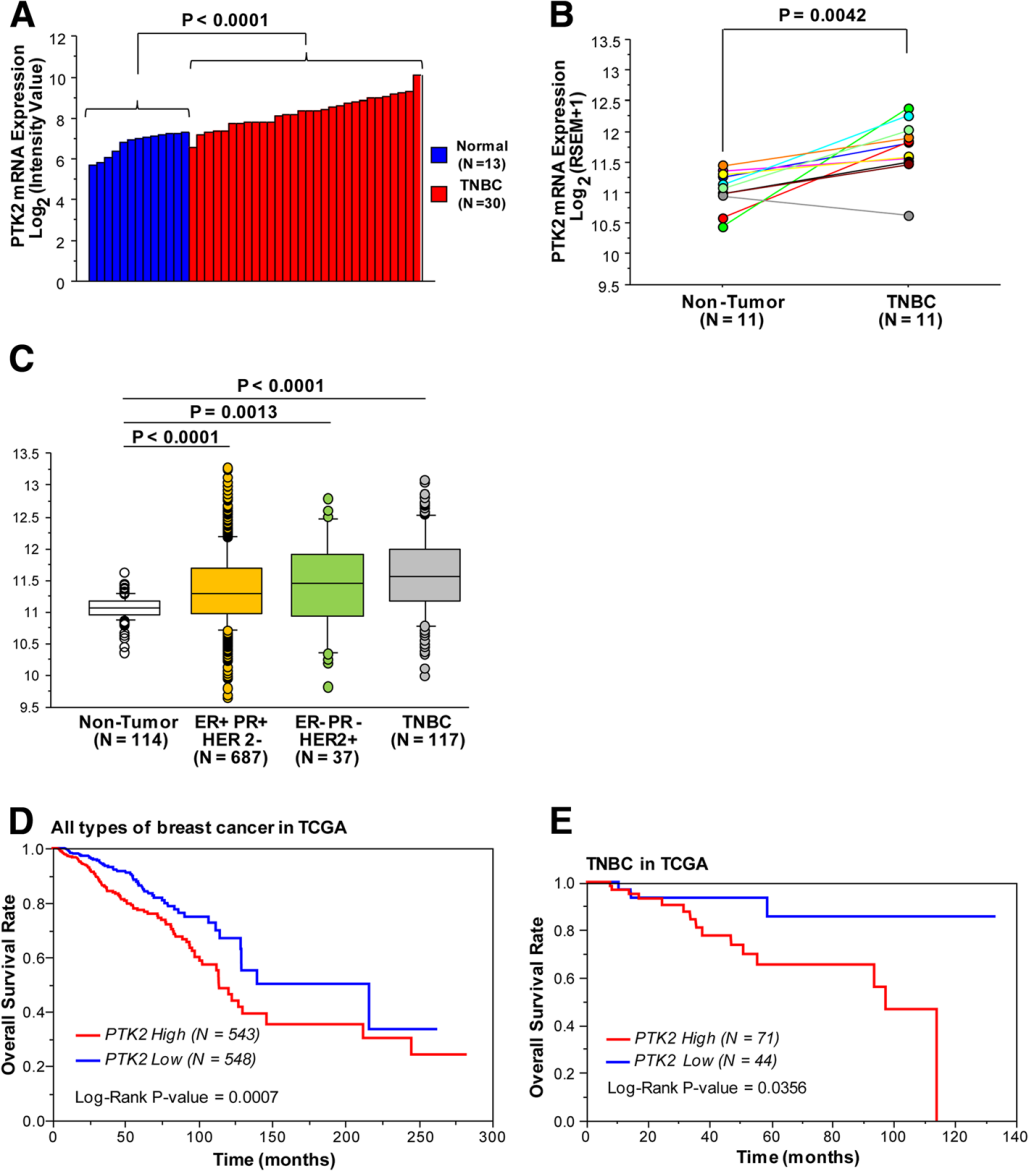
The PTK2 gene encoding FAK is over-expressed in TNBC. **a** Comparison of PTK2 mRNA expression between laser-microbeam microdissected TNBC and normal breast cells. **b** Comparison of PTK2 mRNA expression between matched TNBC and non-tumor breast tissues. **c** Comparison of PTK2 mRNA expression among non-tumor breast tissues, ER+/PR+/HER2-, ER-/PR-/HER2+ and TNBC as reported in TCGA. **d** Clinical outcome in all types of breast cancer with high PTK2 (mRNA Z-score > 1) or low PTK2 (mRNA Z-score ≤ 1) displayed by Kaplan-Meier plots with log-rank tests. **e** Clinical outcome in TNBC patients with high PTK2 (mRNA Z-score > 1) or low PTK2 (mRNA Z-score ≤ 1) displayed by Kaplan-Meier plots with log-rank tests

Previous studies have shown the potential role of FAK toward the breast tumorigenesis and aggressive breast tumor phenotypes [28, 49–51]. On the basis of these findings, we began our study exploring the clinical significance of the FAK encoding gene PTK2 in TNBC by the TCGA database (http://cbioportal.org). The analysis of the RNA sequencing data derived from Invasive Breast Cancer Cohort of TCGA project (The Cancer Genome Atlas: https://cancergenome.nih.gov/), revealed that the PTK2 mRNA expression levels are significantly higher in TNBC compared with normal breast tissues in two independent cohort datasets (Fig. 1a-b). In addition, we found that the PTK2 mRNA expression levels are significantly higher also in ER+/PR+/HER2- and ER-/PR-/HER2+ breast tumors respect to normal breast tissues, however the TNBC samples displayed the highest expression levels among the different breast cancer phenotypes (Fig. 1c). Next, we also assessed the Kaplan–Meier univariate survival of patients groups, comparing those with high PTK2 (mRNA Z-score more than 1) with those exhibiting low PTK2 (mRNA Z-score equal or less than 1). In this regard, we ascertained that the PTK2 high group has a significant poorer overall survival respect to the PTK2 low group in all types of breast cancer as well as in TNBC (Fig. 1d-e). Overall, these data highlight the role of FAK in breast cancer toward the malignant aggressiveness as in TNBC patients.

### GPER mediates FAK activation and the induction of FAs by E2 and G1

**Fig. 2:**
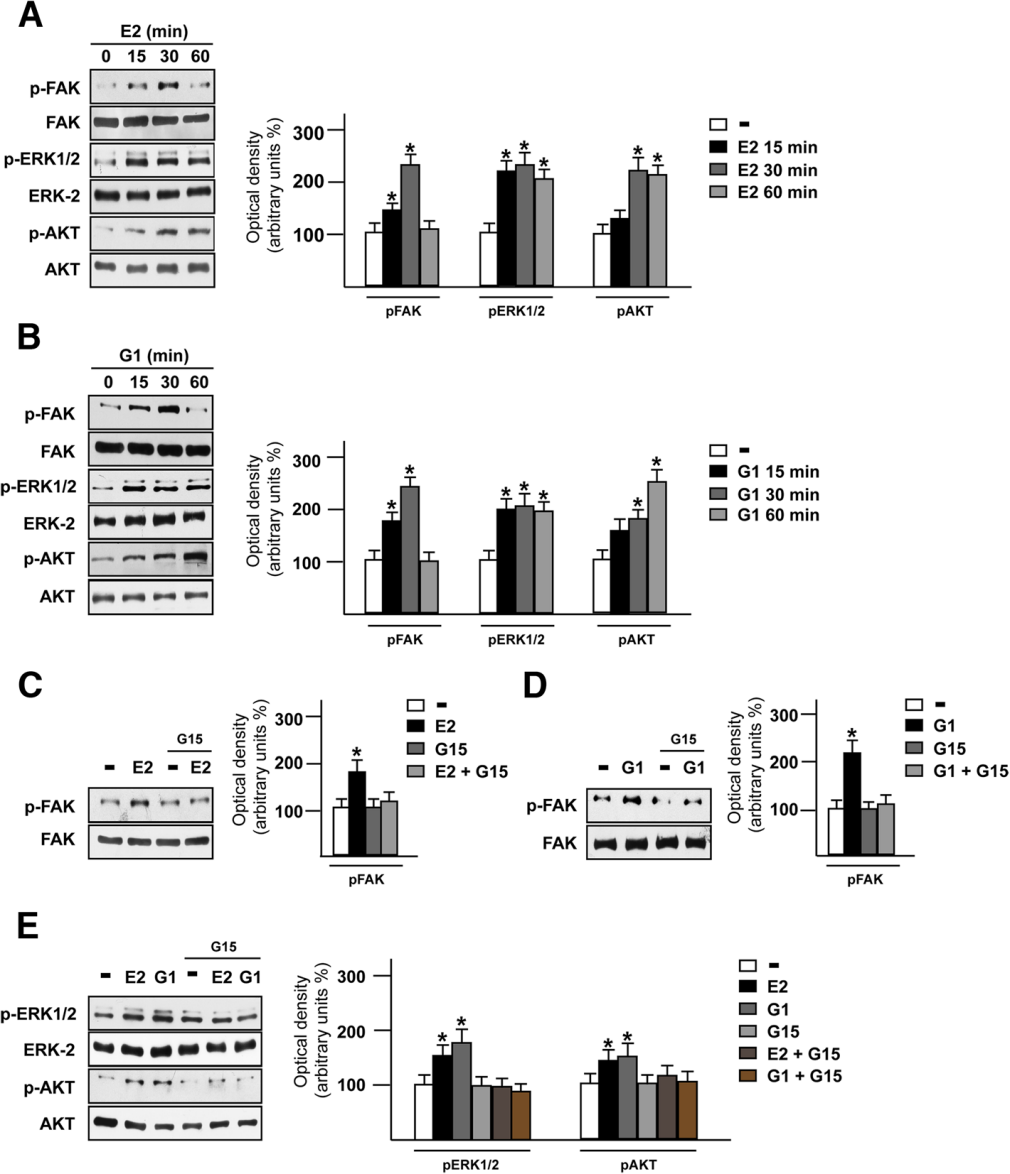
E2 and G1 trigger FAK Y397 activation in TNBC cells. Immunoblots showing FAK, ERK1/2 and AKT phosphorylation upon exposure with 100 nM E2 (**a**) or 100 nM G1 (**b**) in MDA-MB 231 cells, as indicated. Side panels show densitometric analysis of the immunoblots normalized to the loading control. Immunoblots showing FAK phosphorylation in MDA-MB 231 cells treated for 30 min with 100 nM E2 (**c**) or 100 nM G1 (**d**) alone and in combination with 100 nM GPER antagonist G15. Side panels show densitometric analysis of the immunoblots normalized to the loading control. **e** Immunoblots showing ERK1/2 and AKT phosphorylation in MDA-MB 231 cells treated for 30 min with 100 nM E2 or 100 nM G1 alone and in combination with 100 nM GPER antagonist G15. Side panels show densitometric analysis of the immunoblots normalized to the loading control. FAK, ERK-2 and AKT expression levels were used as loading controls for pFAK, pERK1/2 and pAKT, respectively. Results shown are representative of at least three independent experiments. (*) indicates *p* < 0.05

**Fig. 3:**
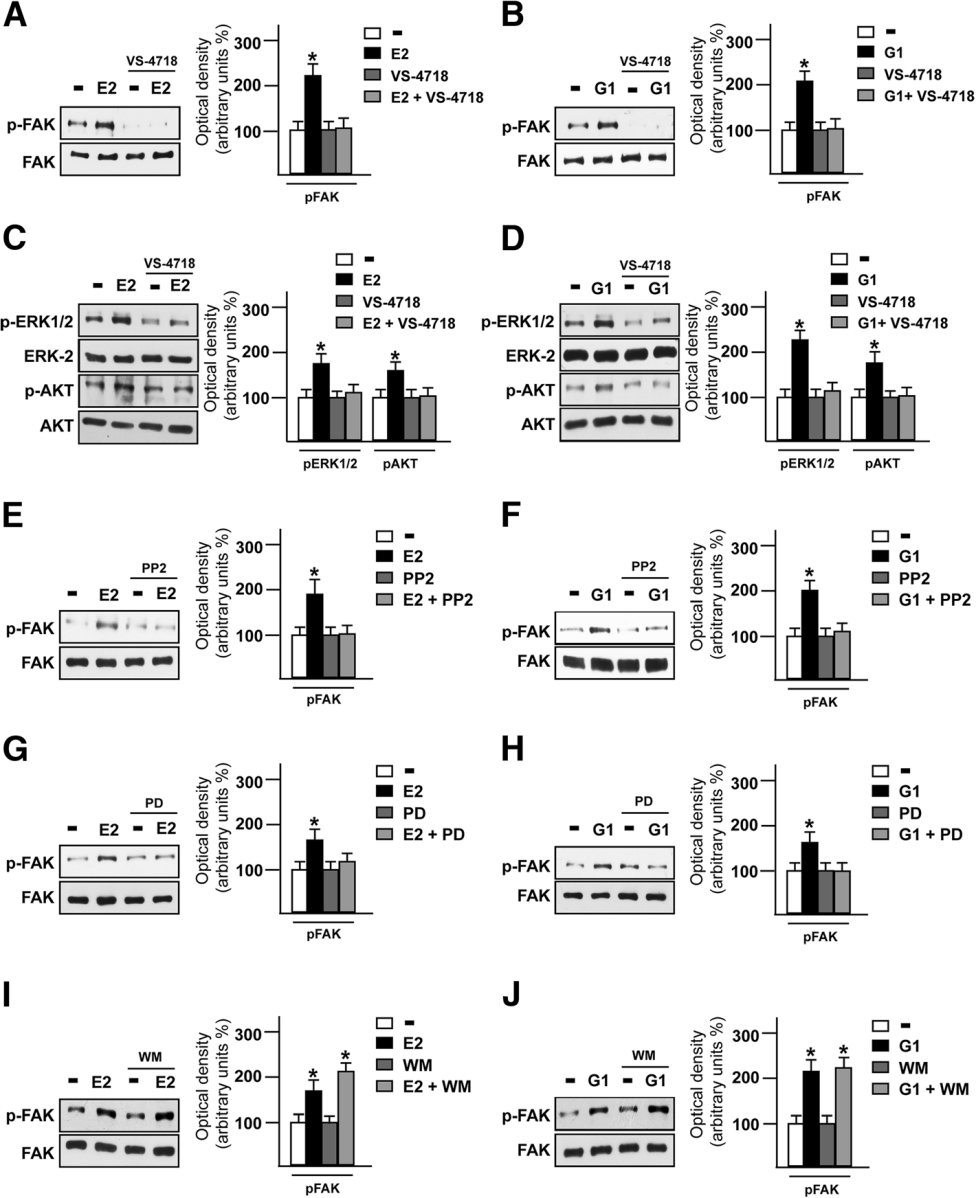
Transduction signaling mediating FAK Y397 phosphorylation. Immunoblots showing FAK phosphorylation in MDA-MB 231 cells treated for 30 min with 100 nM E2 (**a**) and 100 nM G1 (**b**) alone or in combination with 1 μM FAK kinase inhibitor VS-4718. Side panels show densitometric analysis of the immunoblots normalized to the loading control. Immunoblots showing ERK1/2 and AKT phosphorylation in MDA-MB 231 cells treated for 30 min with 100 nM E2 (**c**) and 100 nM G1 (**d**) alone or in combination with 1 μM FAK kinase inhibitor VS-4718. Side panels show densitometric analysis of the immunoblots normalized to the loading control. Immunoblots showing FAK phosphorylation in MDA-MB 231 cells treated for 30 min with 100 nM E2 (**e**) and 100 nM G1 (**f**) alone or in combination with 1 μM c-Src inhibitor PP2. Side panels show densitometric analysis of the immunoblots normalized to the loading control. Immunoblots showing FAK phosphorylation in MDA-MB 231 cells treated for 30 min with 100 nM E2 (**g**) and 100 nM G1 (**h**) alone or in combination with 10 μM MEK inhibitor PD98059 (PD). Side panels show densitometric analysis of the immunoblots normalized to the loading control. Immunoblots showing FAK phosphorylation in MDA-MB 231 cells treated for 30 min with 100 nM E2 (**i**) and 100 nM G1 (**j**) alone or in combination with 10 μM PI3K inhibitor Wortmannin. Side panels show densitometric analysis of the immunoblots normalized to the loading control. FAK, ERK-2 and AKT expression were used as loading controls for pFAK, pERK and pAKT, respectively. Results shown are representative of at least three independent experiments. (*) indicates *p* < 0.05

**Fig. 4:**
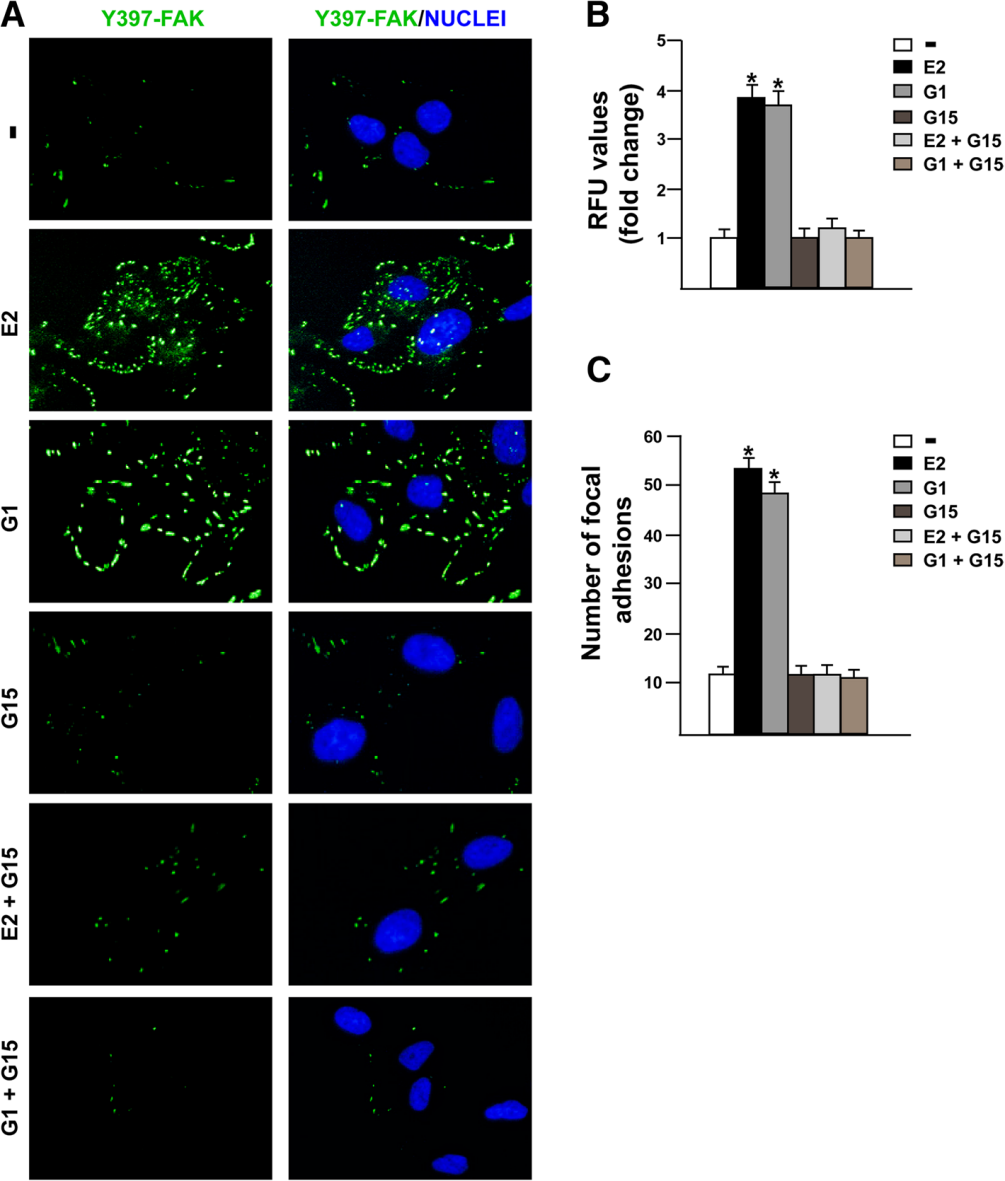
GPER mediates focal adhesions (FAs) in TNBC. **a** Immuofluorescence staining of Focal Adhesions (FAs) in MDA-MB 231 cells treated for 30 min with 100 nM E2 and 100 nM G1 alone or in combination with 100 nM GPER antagonist G15. Cells were probed with anti-phosphotyrosine primary antibody and FITC-conjugated secondary antibody in order to visualize FAs displayed by the green signal, whereas the blue signal indicates the nuclei counterstained with DAPI. Images shown are representative of 10 random fields from three independent experiments. **b** Fluorescence intensities of the green signal were quantified in at least 10 random fields in each condition and results are expressed as fold changes of relative fluorescence units (RFU) upon treatments respect to vehicle-treated cells. **c** FAs number was quantified in at least 10 random fields in each condition and results are expressed as mean focal adhesions ± SD from three independent experiments upon treatments respect to vehicle-treated cells. (*) indicates *p* < 0.05

FAK represents a main component in the integrins-mediated transduction pathway and contributes to diverse signaling cascades triggered by a wide range of stimuli as growth factors, cytokines and G-protein coupled receptor agonists [52, 53]. As previous studies have revealed that estrogens may regulate the focal adhesion complexes not only through the classical estrogen receptor α (ERα) in breast tumor and endothelial cells [54–56] but also via GPER in human dermal fibroblasts [57], we aimed to investigate whether GPER is involved in the activation of FAK in TNBC MDA-MB 231 and SUM159 cells [58]. Both E2 and the GPER selective agonist G1 triggered the Y397 FAK phosphorylation along with the activation of ERK1/2 and AKT in MDA-MB 231 cells (Fig. 2a-b), however these responses were no longer evident in the presence of the GPER antagonist G-15 (Fig. 2c-e) or using the FAK inhibitor namely VS-4718 (also known as PND-1186) [34] (Fig. 3a-d). Likewise, we ascertained that both the GPER antagonist G-15 and the FAK kinase inhibitor VS-4718 prevent the Y397 FAK phosphorylation induced by E2 and G1 in SUM159 TNBC cells (Additional file 1: Figure S1A-D). Next, we assessed that the c-Src kinase inhibitor PP2 and the MEK inhibitor PD98059, but not the PI3K inhibitor wortmannin, abolish the Y397 FAK phosphorylation upon E2 and G1 exposure (Fig. 3e-j). As expected, the MEK inhibitor PD98059 and the PI3K inhibitor wortmannin, inhibited respectively the phosphorylation of ERK and AKT induced by E2 and G1 (Additional file 2: Figure S2A-D). Overall, these findings point out that FAK activation by estrogenic signaling may occur through the GPER/c-Src/MEK transduction pathway. As FAs are important sub-cellular structure mediating cell adhesion to ECM in tumor spreading [59, 60], we then determined by immunofluorescence assays that FAs formation prompted by E2 and G1 is prevented using the GPER antagonist G-15 (Fig. 4a-c), thus suggesting the involvement of GPER in the above mentioned response observed in MDA-MB 231 cells.

### FAK is involved in the STAT3 nuclear accumulation and gene expression changes induced by E2 and G1 through GPER

**Fig. 5:**
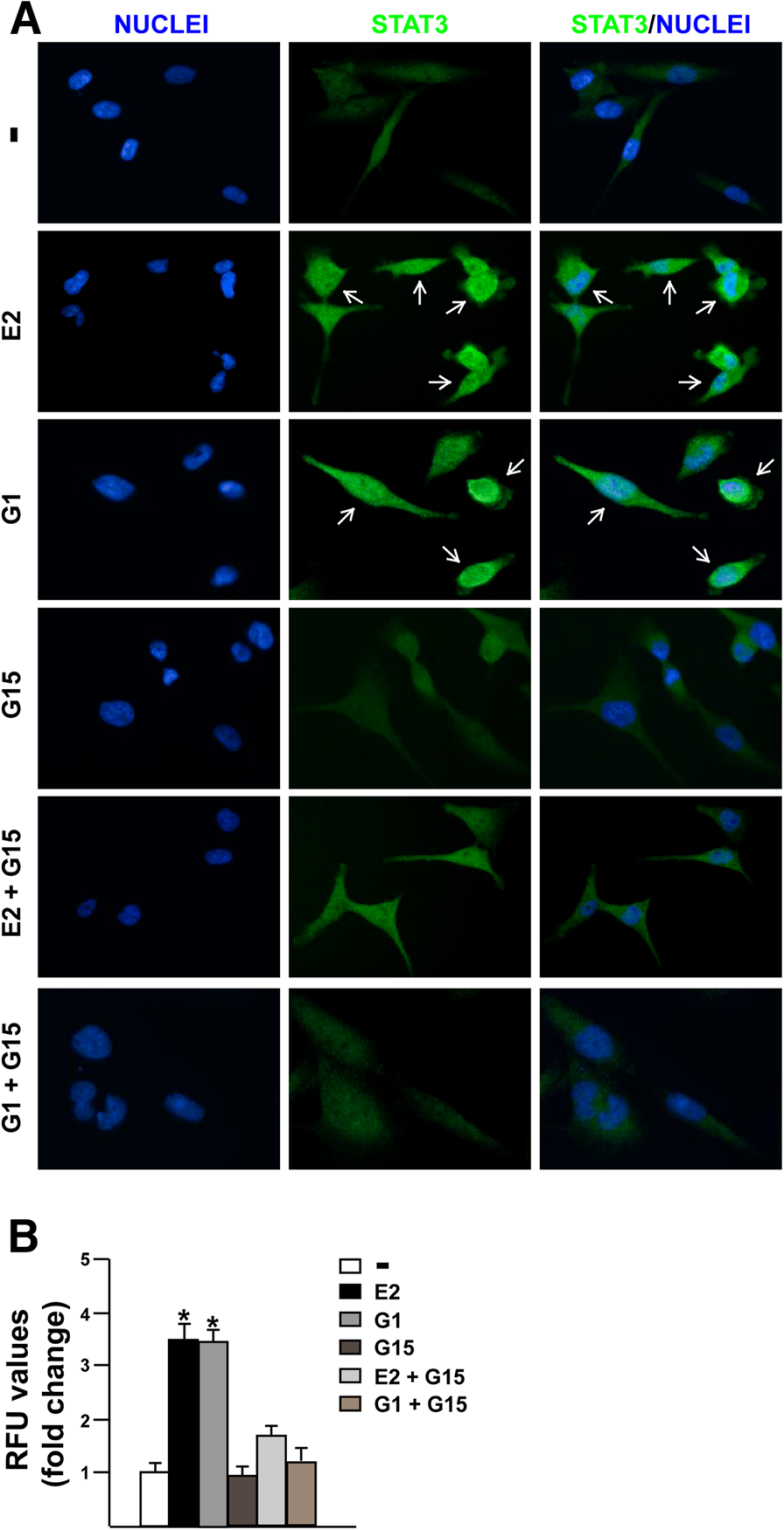
The GPER antagonist G-15 reduces STAT3 nuclear accumulation triggered by estrogens. **a** Immunofluorescence staining of STAT3 in MDA-MB 231 cells treated for 1 h with 100 nM E2 and 100 nM G1 alone or in combination with 100 nM GPER antagonist G-15. Cells were probed with rabbit anti-STAT3 primary antibody followed by FITC-conjugated secondary antibody in order to detect STAT3 displayed by the green signal, whereas the blue signal indicates the nuclei counterstained with DAPI. Images shown are representative of 10 random fields. **b** Fluorescence intensities of the green signal were quantified in at least 10 random fields in each condition from three independent experiments and data are expressed as fold changes of relative fluorescence units (RFU) upon treatments respect to vehicle-treated cells. Arrows indicate STAT3 nuclear accumulation. (*) indicates *p* < 0.05

**Fig. 6:**
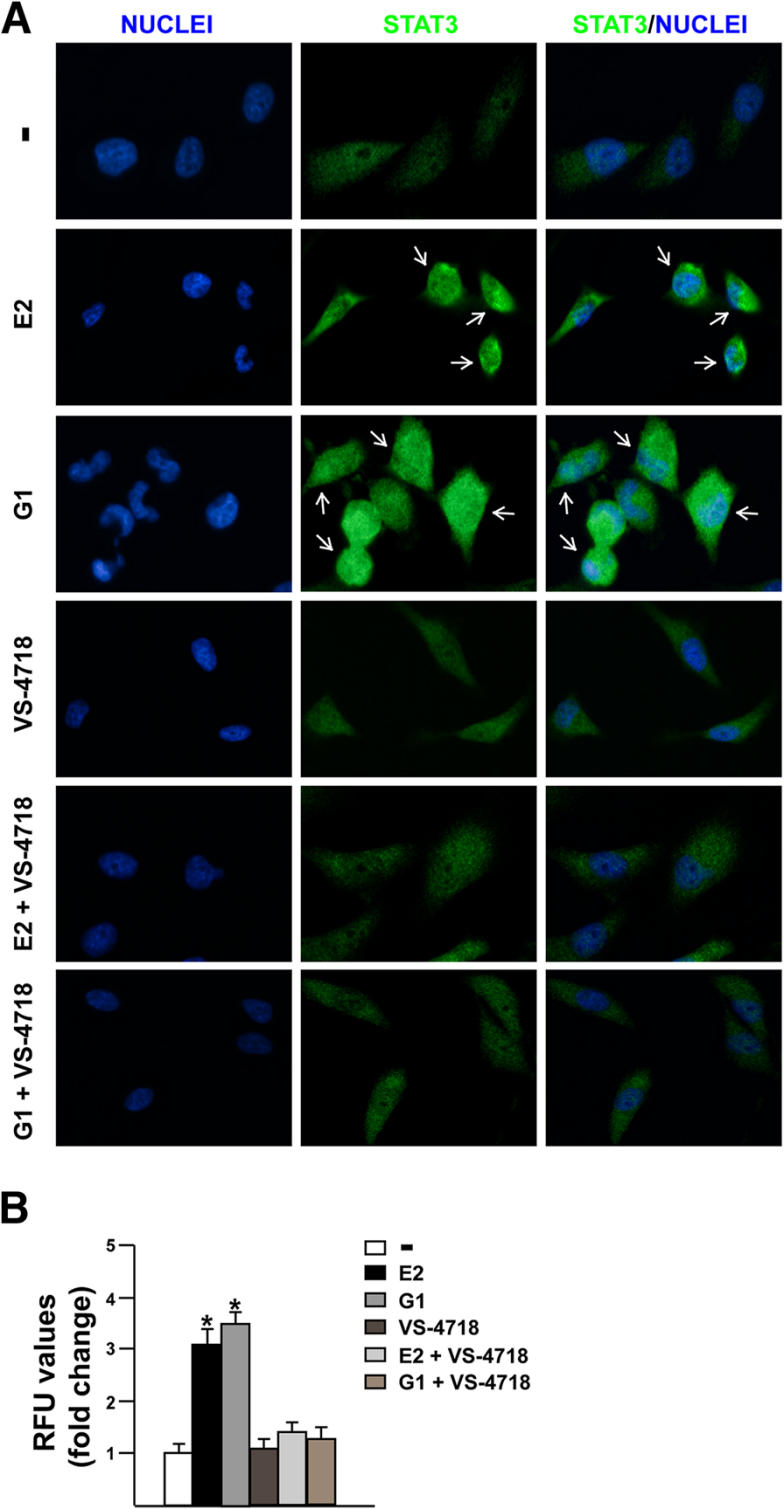
The FAK inhibitor VS-4718 prevents STAT3 nuclear accumulation triggered by estrogens. **a** Immunofluorescence staining of STAT3 in MDA-MB 231 cells treated for 1 h with 100 nM E2 and 100 nM G1 alone or in combination with 1 μM FAK kinase inhibitor VS-4718. Cells were probed with rabbit anti-STAT3 primary antibody followed by FITC-conjugated secondary antibody in order to detect STAT3 displayed by the green signal, whereas the blue signal indicates the nuclei counterstained with DAPI. Images shown are representative of 10 random fields. **b** Fluorescence intensities of the green signal were quantified in at least 10 random fields in each condition from three independent experiments and data are expressed as fold changes of relative fluorescence units (RFU) upon treatments respect to vehicle-treated cells. Arrows indicate STAT3 nuclear accumulation. (*) indicates *p* < 0.05

**Fig. 7:**
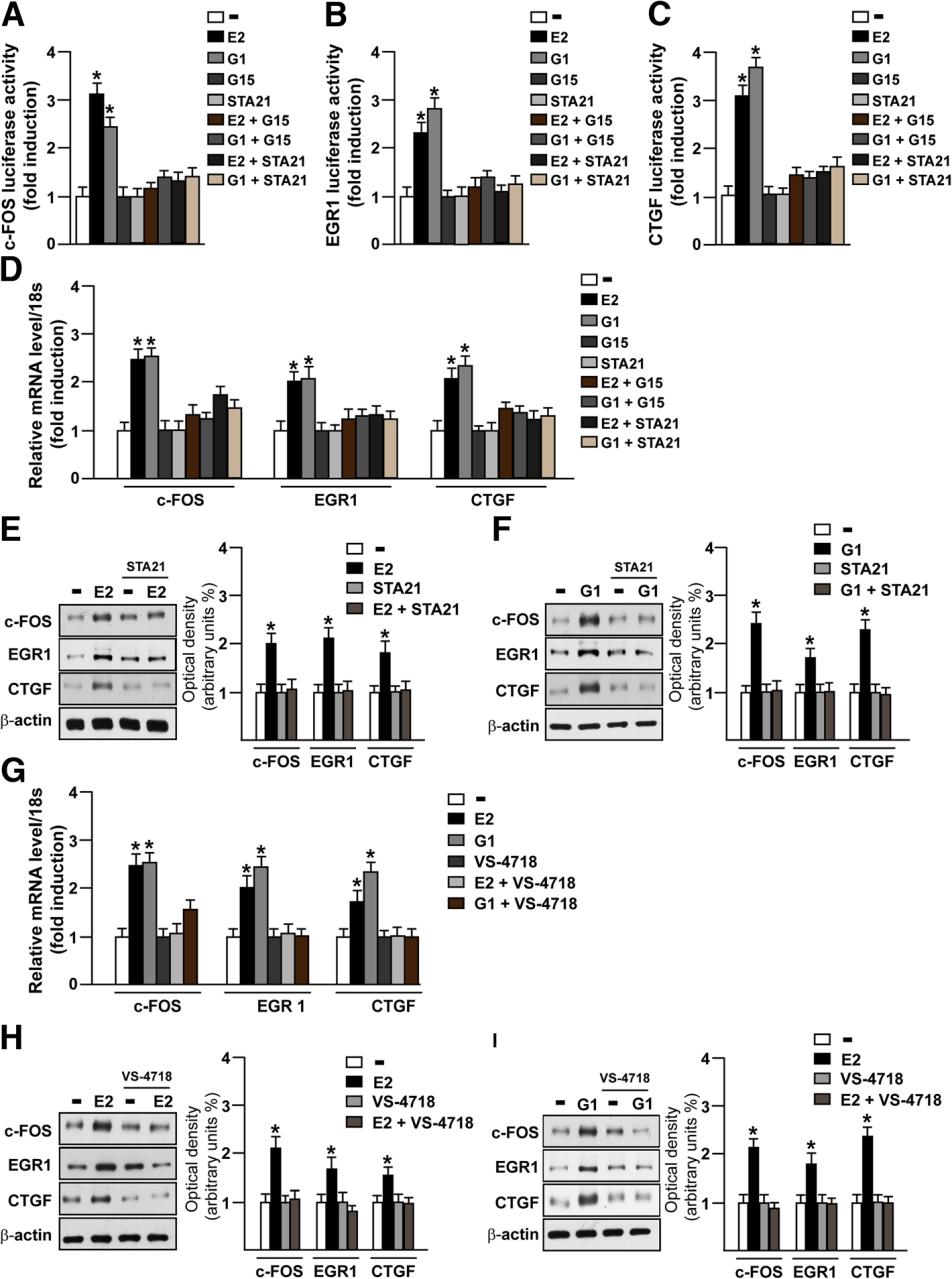
c-FOS, EGR1 and CTGF regulation by FAK and STAT3. c-FOS (**a**), EGR1 (**b**) and CTGF (**c**) luciferase promoter activity in MDA-MB 231 cells treated for 18 h with 100 nM E2 and 100 nM G1 alone or in combination with 100 nM GPER antagonist G15 or 20 μM STAT3 inhibitor STA21. The luciferase activities were normalized to the internal transfection control and values of cells receiving vehicle were set as 1-fold induction upon which the activities induced by treatments were calculated. Each data point represents the mean ± SD of three independent experiments performed in triplicate. **d** c-FOS, EGR1 and CTGF mRNA expression measured by real time-PCR in MDA-MB 231 cells treated for 4 h with 100 nM E2 and 100 nM G1 alone or in combination with 100 nM GPER antagonist G15 or 20 μM STAT3 inhibitor STA21. Values normalized to the 18 s expression are shown as fold changes of the mRNA expression induced by treatments compared to cells treated with vehicle (−). **e-f** Immunoblots showing c-FOS, EGR1 and CTGF protein expression in MDA-MB 231 cells treated for 4 h with 100 nM E2 (**e**) and 100 nM G1 (**f**) alone or in combination with 20 μM STAT3 inhibitor STA21. Side panels show densitometric analysis of the immunoblots normalized to β-actin. **g** c**-**FOS, EGR1 and CTGF mRNA expression measured by real time-PCR in MDA-MB 231 cells treated for 4 h with 100 nM E2 and 100 nM G1 alone or in combination with 1 μM FAK kinase inhibitor VS-4718. Values normalized to the 18 s expression are shown as fold changes of the mRNA expression induced by treatments compared to cells treated with vehicle (−). Immunoblots showing c-FOS, EGR1 and CTGF protein expression in MDA-MB 231 cells treated for 4 h with 100 nM E2 (**h**) and 100 nM G1 (**i**) alone or in combination with 1 μM FAK kinase inhibitor VS-4718. Side panels show densitometric analysis of the immunoblots normalized to β-actin. Results shown are representative of three independent experiments. (*) indicates *p* < 0.05

It has been reported that FAK knockdown may affect the activation of the signal transducer and activator of transcription 3 (STAT3), which is a point of convergence for numerous oncogenic pathways [61–65]. As GPER was also involved in the activation of STAT3 [66, 67], we aimed to evaluate the role of FAK in the STAT3 nuclear accumulation triggered by estrogenic GPER signaling. Of note, we found that E2 and G1 induce the nuclear shuttle of STAT3, however this effect was no longer evident in the presence of the GPER antagonist G-15 (Fig. 5a-b) or using the FAK inhibitor VS-4718 (Fig. 6a-b), as assessed by immunofluorescence assay in MDA-MB 231 cells. As our and other previous studies have evidenced that GPER triggers a specific gene signature in breast cancer cells toward relevant biological effects [45, 68, 69], we then sought to investigate whether STAT3 may contribute to gene expression changes mediated by GPER in MDA-MB 231 cells. First, we assessed that the GPER antagonist G-15 and the STAT3 inhibitor namely STA21 repress the transactivation of c-FOS (Fig. 7a), EGR1 (Fig. 7b) and CTGF (Fig. 7c) promoter activity triggered by E2 and G1 treatments. In accordance with these results, G-15 and STA21 reduced the mRNA expression levels of c-FOS, EGR1 and CTGF induced by E2 and G1 (Fig. 7d). Interestingly, c-FOS, EGR1 and CTGF protein levels induced by E2 and G1 were abrogated using STA21 (Fig. 7e-f) and both the mRNA and protein levels of c-FOS, EGR1 and CTGF triggered by E2 and G1 were prevented using the FAK inhibitor VS-4718 (Fig. 7g-i). Altogether**,** these results reveal that STAT3 along with FAK may contribute to the regulation of GPER target genes in TNBC cells.

### FAK and STAT3 inhibition prevents the migration of TNBC cells

**Fig. 8:**
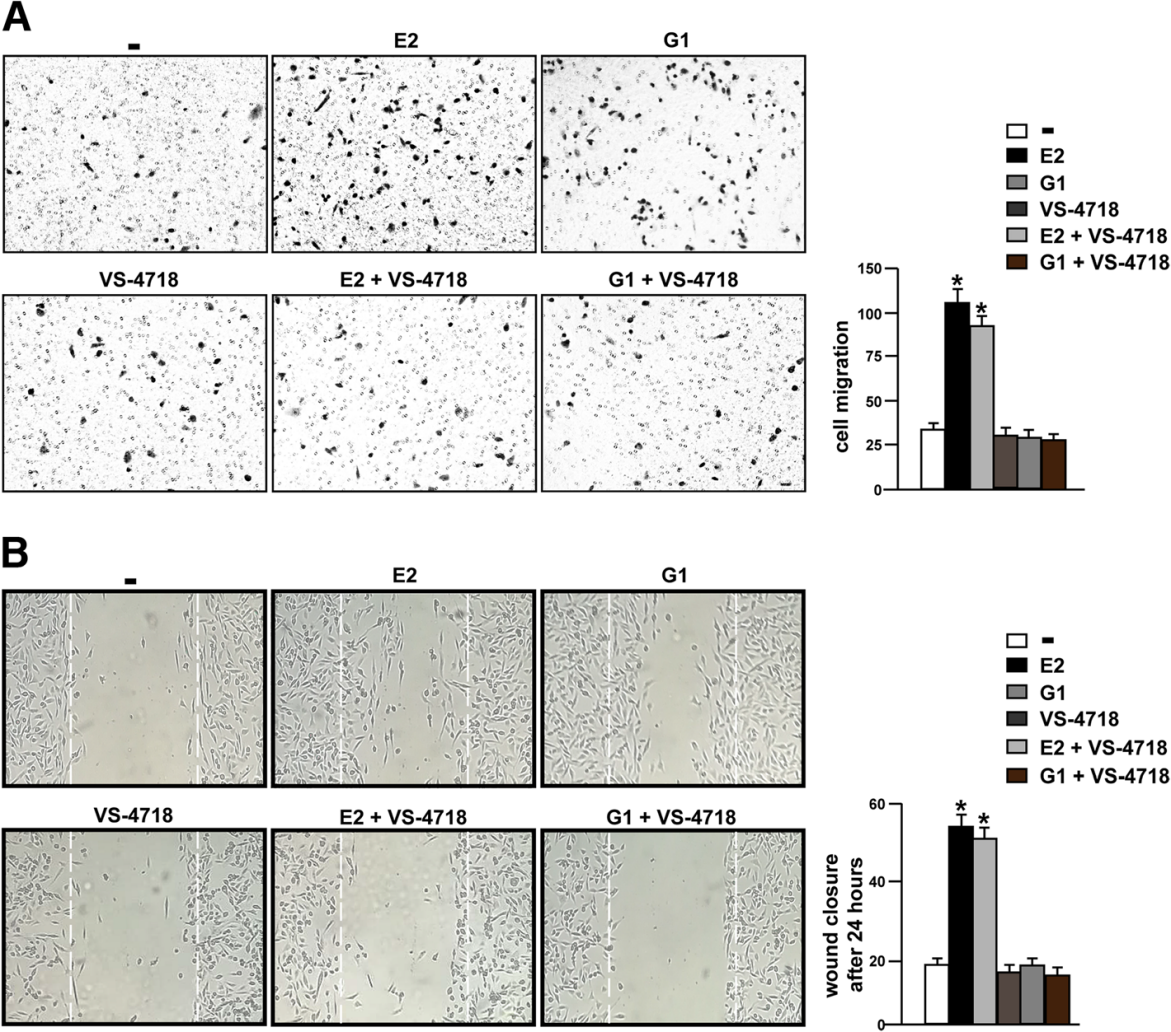
The FAK inhibitor VS-4718 inhibits the migration of TNBC cells induced by E2 and G1. **a** Boyden Chamber assays showing the migration of MDA-MB 231 cells treated for 4 h with 100 nM E2 and 100 nM G1 alone or in combination with 1 μM FAK kinase inhibitor VS-4718. The results are shown as cells migrating through the membrane at the bottom of the well upon treatments respect to vehicle (−). Results shown are representative of three independent experiments. **b** Cell migration was evaluated by wound-healing assay in MDA-MB 231 cells treated for 24 h with 100 nM E2 and 100 nM G1 alone or in combination with 1 μM FAK kinase inhibitor VS-4718. White dotted lines indicate the wound borders at the beginning of the assay and recorded 24 h post- scratching. Results shown are representative of three independent experiments. (*) indicates *p* < 0.05

**Fig. 9:**
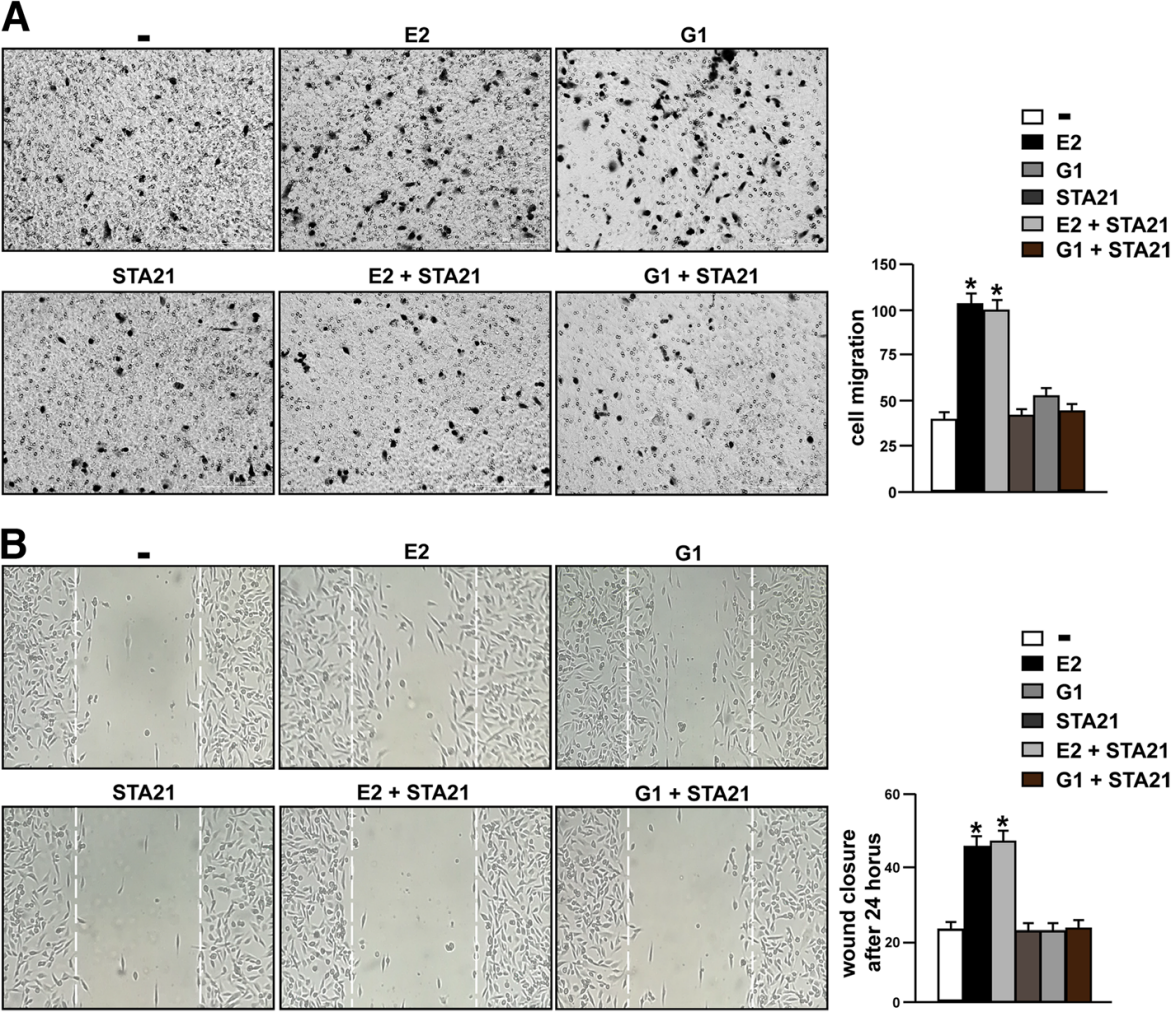
The STAT3 inhibitor STA21 suppresses the migration of TNBC cells induced by E2 and G1. **a** Boyden Chamber assays showing the migration of MDA-MB 231 cells treated for 4 h with 100 nM E2 and 100 nM G1 alone or in combination with 20 μM STAT3 inhibitor STA21. The results are shown as cells migrating through the membrane at the bottom of the well upon treatments respect to vehicle (−). Results shown are representative of three independent experiments. **b** Cell migration was evaluated by wound-healing assay in MDA-MB 231 cells treated for 24 h with 100 nM E2 and 100 nM G1 alone or in combination with 20 μM STAT3 inhibitor STA21. White dotted lines indicate the wound borders at the beginning of the assay and recorded 24 h post- scratching. Results shown are representative of three independent experiments. (*) indicates *p* < 0.05

Several reports have highlighted the role of FAK in the migration of cancer cells [70]. Accordingly, we assessed that the migratory effects induced by E2 and G1 were abolished not only in the presence of the GPER antagonist G-15 (Additional file 3: Figure S3A) but also using the FAK inhibitor VS-4718, as evaluated by boyden chamber assay performed in MDA-MB 231 cells (Fig. 8a). In addition, scratch monolayer experiments evidenced that G-15 (Additional file 3: Figure S3B) and VS-4718 (Fig. 8b) lessen the wound closure triggered by E2 and G1. In order to further corroborate these results, we assessed that both the GPER antagonist G-15 and the FAK inhibitor VS-4718 reduce the migration of SUM159 cells stimulated by E2 and G1 (Additional file 4: Figure S4A). As STAT3 may contribute to the migration of breast cancer cells [71], we aimed to ascertain its involvement in the migratory features of TNBC cells mediated by GPER. Boyden chamber and wound healing assays revealed that the migration of MDA-MB 231 cells stimulated by E2 and G1 is abolished using the STAT3 inhibitor STA21 (Fig. 9a-b). Overall, both FAK and STAT3 may contribute to the invasive skills of TNBC cells prompted by estrogenic GPER signaling.

## Discussion

In the present study, we first assessed that the mRNA expression of PTK2/FAK is associated with worse survival rates and up-regulated in the aggressive TNBC respect to non-TNBC and normal breast samples, as determined by a bioinformatic analysis of cancer genomics TCGA datasets (www.cbioportal.org). Next, to provide novel insights on the molecular mechanisms through which FAK may be involved in the TNBC progression, we ascertained its role in gene expression changes and the migratory skills of TNBC cells triggered by estrogenic GPER signaling. In particular, we found that GPER stimulation induces Y397 FAK phosphorylation and increases the number of FAs in TNBC cells. In addition, we demonstrated the role exerted by FAK in the GPER-mediated nuclear accumulation of STAT3 and the involvement of both FAK and STAT3 toward the regulation of GPER target genes and the migratory responses of TNBC cells.

Several studies have correlated FAK expression and activity with different types of primary and metastatic cancers, including breast malignancy [36, 72]. In this regard, FAK expression was shown not only associated with invasive and metastatic breast cancer [73], but also as an early event occurring in breast tumorigenesis [51, 74]. In accordance with these studies, the expression of FAK was linked to a poor clinical outcome [31], therefore further highlighting the contribution of FAK in the development of breast tumor.

It has been established that FAK plays a main action in the formation of focal adhesion complexes, hence acting as a key regulator of important processes in both normal and cancer cells [75, 76]. A well characterized mechanism promoting FAK activation involves the integrin-ECM engagment and the subsequent co-clustering of proteins (i.e. talin and paxillin) with the cytoplasmic tail of integrin [77]. In addition, FAK may be activated by various extracellular stimuli such as steroids like estrogens [56], growth factors [78], cytokines [79], phospholipids, lipid mediators [80] and GPCRs initiated pathways [12, 81].

Estrogens may be involved in the regulation of several cytoskeletal and membrane remodeling components as the focal adhesion complexes [54, 55]. In particular, estrogens regulate cell morphology and the interaction with ECM, thus driving the cell movement under the control of the actin organization [54, 55]. In this regard, it has been reported that estrogens through ERα induce the phosphorylation of FAK and its subsequent translocation within the membrane sites where focal adhesion complexes are assembled [56]. Besides, estrogens-induced cytoskeleton re-organization and focal adhesion strengthening may also occur via GPER [57], which mediates estrogenic signaling in diverse types of tumors [44, 68, 82–84]. Further extending the aforementioned findings, in the present study we have documented that estrogens through GPER triggers the activation of FAK in TNBC cells, in accordance with previous data obtained in different cancer cell contexts [85].

Estrogenic GPER signaling may contribute to the regulation of several genes in tumor cells via diverse transcription factors [45] as well as the involvement of STAT3 [66, 67]. Upon activation STAT3 forms homo- or heterodimers through the SH2 and the C-terminal domains, then translocates into the nucleus where it binds specific sequences located in the promoter sequences of target genes [61, 86]. In this scenario, our immunofluorescence studies revealed that GPER mediates an enhanced nuclear accumulation of STAT3 in TNBC cells. Interestingly, this effect was prevented not only in the presence of the GPER antagonist G-15, but also using the FAK inhibitor VS-4718, in accordance with previous studies suggesting that FAK is involved in the STAT3 activation and transcriptional activity [62–65]. Of note, not only the GPER antagonist G15 but also the DNA-binding STAT3 inhibitor STA21 reduced the promoter activity and the expression of the GPER target genes c-FOS, EGR1 and CTGF [45], further corroborating the involvement of STAT3 in the regulation of these genes [87–89]. Likewise, the expression levels of c-FOS, EGR1 and CTGF were reduced using the FAK inhibitor VS-4718, thus suggesting that FAK is also involved in the GPER-mediated regulation of the aforementioned genes.

FAK signaling has long been linked to the cell migration process, which represents a crucial skill toward cancer cell invasion and metastasis [90]. Indeed, several FAK-downstream pathways have been implicated in cell migration as Src and PI3K transduction cascades [91–95]. In addition, FAK-mediated cell migration was shown to require diverse key factors involved in the cytoskeleton remodeling as the Rho subfamily of small GTPases [96], N-WASP [97], and Arp2/3 complex [98]. On the basis of these observations, FAK inhibitors are currently considered promising chemoterapeutic agents [8]. In this respect, our data further highlight the use of FAK inhibitors given that the treatments with VS-4718 prevented the migration of TNBC cells upon the agonist activation of GPER. Overall, our findings suggest that FAK is involved in the stimulatory action of GPER in TNBC cells, however further investigations are needed to better define this functional cooperation toward the aggressive features of breast malignancy.

## Conclusion

In the present study we have provided new evidence regarding the engagement of FAK in the estrogenic GPER signaling in TNBC cells. In particular, we have assessed that FAK contributes to the GPER mediated STAT3 activation, the gene expression changes and the invasiveness of TNBC cells. Together, these findings suggest that the action of GPER through FAK may be considered toward combination treatments targeting TNBC.

## Additional files

Additional file 1: **Figure S1.** GPER stimulation triggers FAK Y397 activation in SUM159 TNBC cells. Immunoblots showing FAK phosphorylation in SUM159 cells treated for 30 min with 100 nM E2 (A) or 100 nM G1 (B) alone or in combination with 100 nM GPER antagonist G-15. Side panels show densitometric analysis of the immunoblots normalized to the loading control. Immunoblots showing FAK phosphorylation in SUM159 cells treated for 30 min with 100 nM E2 (C) or 100 nM G1 (D) alone and in combination with 1 μM FAK kinase inhibitor VS-4718. Side panels show densitometric analysis of the immunoblots normalized to the loading control. FAK expression was used as loading control for pFAK. Results shown are representative of at least three independent experiments. (*) indicates *p* < 0.05 (TIF 1732 kb)

Additional file 2: **Figure S2.** The MEK inhibitor PD98059 and the PI3K inhibitor Wortmannin prevent respectively the activation of ERK and AKT induced by E2 and G1 in MDA-MB 231 TNBC cells. Immunoblots showing ERK phosphorylation in MDA-MB 231 cells treated for 30 min with 100 nM E2 (A) or 100 nM G1 (B) alone or in combination with 10 μM MEK inhibitor PD98059 (PD). Side panels show densitometric analysis of the immunoblots normalized to the loading control. Immunoblots showing AKT phosphorylation in MDA-MB 231 cells treated for 30 min with 100 nM E2 (C) or 100 nM G1 (D) alone and in combination with 10 μM PI3K inhibitor Wortmannin. Side panels show densitometric analysis of the immunoblots normalized to the loading control. ERK and AKT expression levels were used as loading controls for pERK and pAKT. Results shown are representative of at least three independent experiments. (*) indicates *p* < 0.05 (TIF 1738 kb)

Additional file 3: **Figure S3.** The GPER antagonist G-15 reduces the migration of MDA-MB 231 TNBC cells induced by E2 and G1. (A) Boyden Chamber assays showing the migration of MDA-MB 231 cells treated for 4 h with 100 nM E2 and 100 nM G1 alone or in combination with 100 nM GPER antagonist G-15. The results are shown as cells migrating through the membrane at the bottom of the well upon treatments respect to vehicle (−). Results shown are representative of three independent experiments. (B) Cell migration was evaluated by wound-healing assay in MDA-MB 231 cells treated for 24 h with 100 nM E2 and 100 nM G1 alone or in combination with 100 nM GPER antagonist G-15. White dotted lines indicate the wound borders at the beginning of the assay and recorded 24 h post-scratching. Results shown are representative of three independent experiments. (*) indicates *p* < 0.05

Additional file 4: **Figure S4.** The GPER antagonist G-15 and the FAK inhibitor VS-4718 inhibit the migration of SUM159 TNBC cells induced by E2 and G1. (A) Boyden Chamber assays showing the migration of SUM159 cells treated for 4 h with 100 nM E2 and 100 nM G1 alone or in combination with 100 nM GPER antagonist G-15 and 1 μM FAK kinase inhibitor VS-4718. The results are shown as cells migrating through the membrane at the bottom of the well upon treatments respect to vehicle (−). Results shown are representative of three independent experiments. (*) indicates *p* < 0.05

## Abbreviations

FAK: Focal adhesion kinase
GPER: G-coupled protein estrogen receptor
STA21: STAT3 inhibitor
STAT3: Signal transducer and activator of transcription 3
TNBC: Triple negative breast cancer
VS-4718: FAK inhibitor
