# Supplementary figures and images for: Focal adhesion kinase (FAK) activation by estrogens involves GPER in triple-negative breast cancer cells
Source: J Exp Clin Cancer Res. 2019 Feb 6;38:58. doi: 10.1186/s13046-019-1056-8 (PMC6364402; doi:10.1186/s13046-019-1056-8)

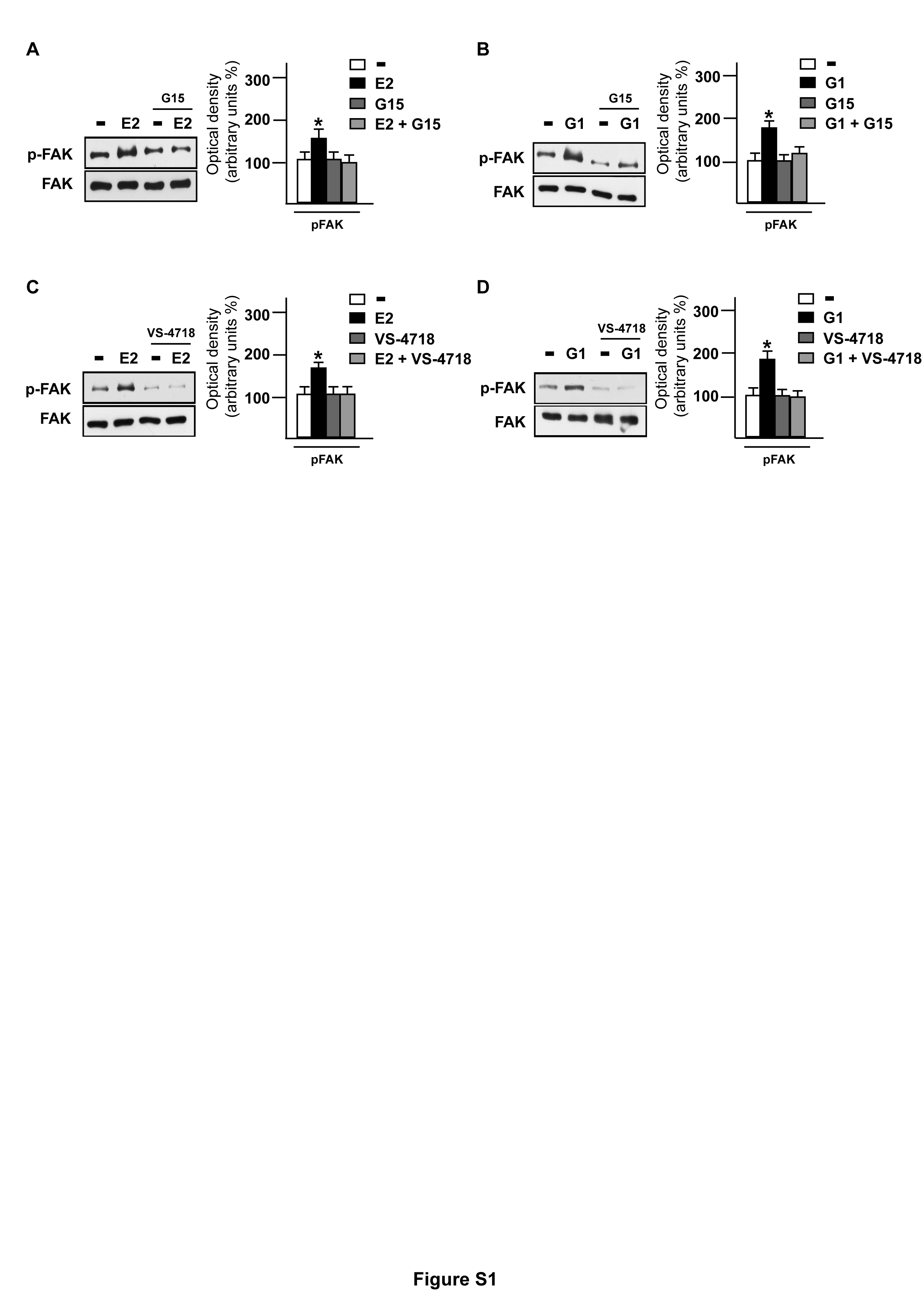

Supplement: Supplementary file 1 — Figure S1. GPER stimulation triggers FAK Y397 activation in SUM159 TNBC cells. Immunoblots showing FAK phosphorylation in SUM159 cells treated for 30 min with 100 nM E2 (A) or 100 nM G1 (B) alone or in combination with 100 nM GPER antagonist G-15. Side panels show densitometric analysis of the immunoblots normalized to the loading control. Immunoblots showing FAK phosphorylation in SUM159 cells treated for 30 min with 100 nM E2 (C) or 100 nM G1 (D) alone and in combination with 1 μM FAK kinase inhibitor VS-4718. Side panels show densitometric analysis of the immunoblots normalized to the loading control. FAK expression was used as loading control for pFAK. Results shown are representative of at least three independent experiments. (*) indicates p < 0.05 (TIF 1732 kb) [file 13046_2019_1056_MOESM1_ESM.tif]

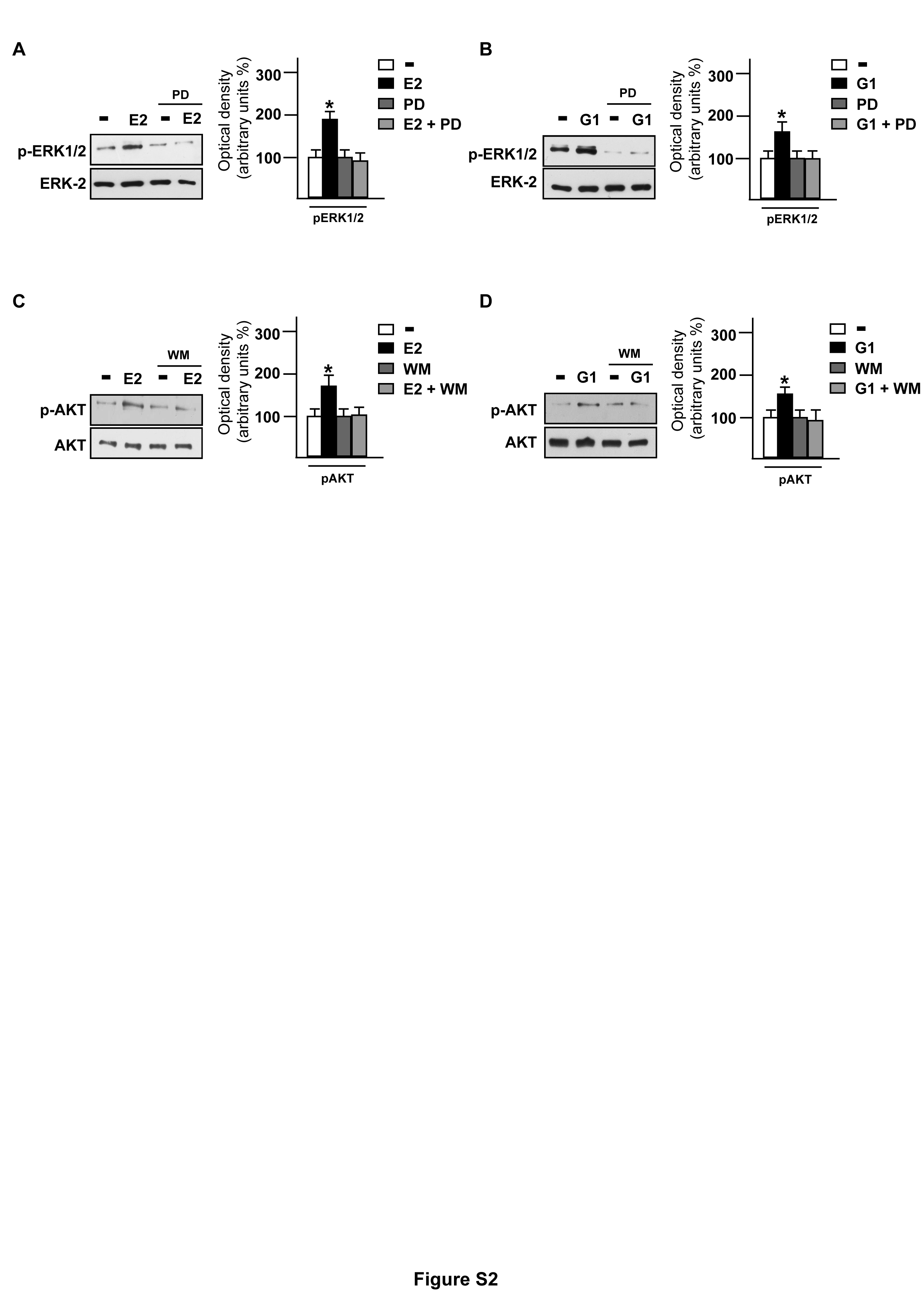

Supplement: Supplementary file 2 — Figure S2. The MEK inhibitor PD98059 and the PI3K inhibitor Wortmannin prevent respectively the activation of ERK and AKT induced by E2 and G1 in MDA-MB 231 TNBC cells. Immunoblots showing ERK phosphorylation in MDA-MB 231 cells treated for 30 min with 100 nM E2 (A) or 100 nM G1 (B) alone or in combination with 10 μM MEK inhibitor PD98059 (PD). Side panels show densitometric analysis of the immunoblots normalized to the loading control. Immunoblots showing AKT phosphorylation in MDA-MB 231 cells treated for 30 min with 100 nM E2 (C) or 100 nM G1 (D) alone and in combination with 10 μM PI3K inhibitor Wortmannin. Side panels show densitometric analysis of the immunoblots normalized to the loading control. ERK and AKT expression levels were used as loading controls for pERK and pAKT. Results shown are representative of at least three independent experiments. (*) indicates p < 0.05 (TIF 1738 kb) [file 13046_2019_1056_MOESM2_ESM.tif]

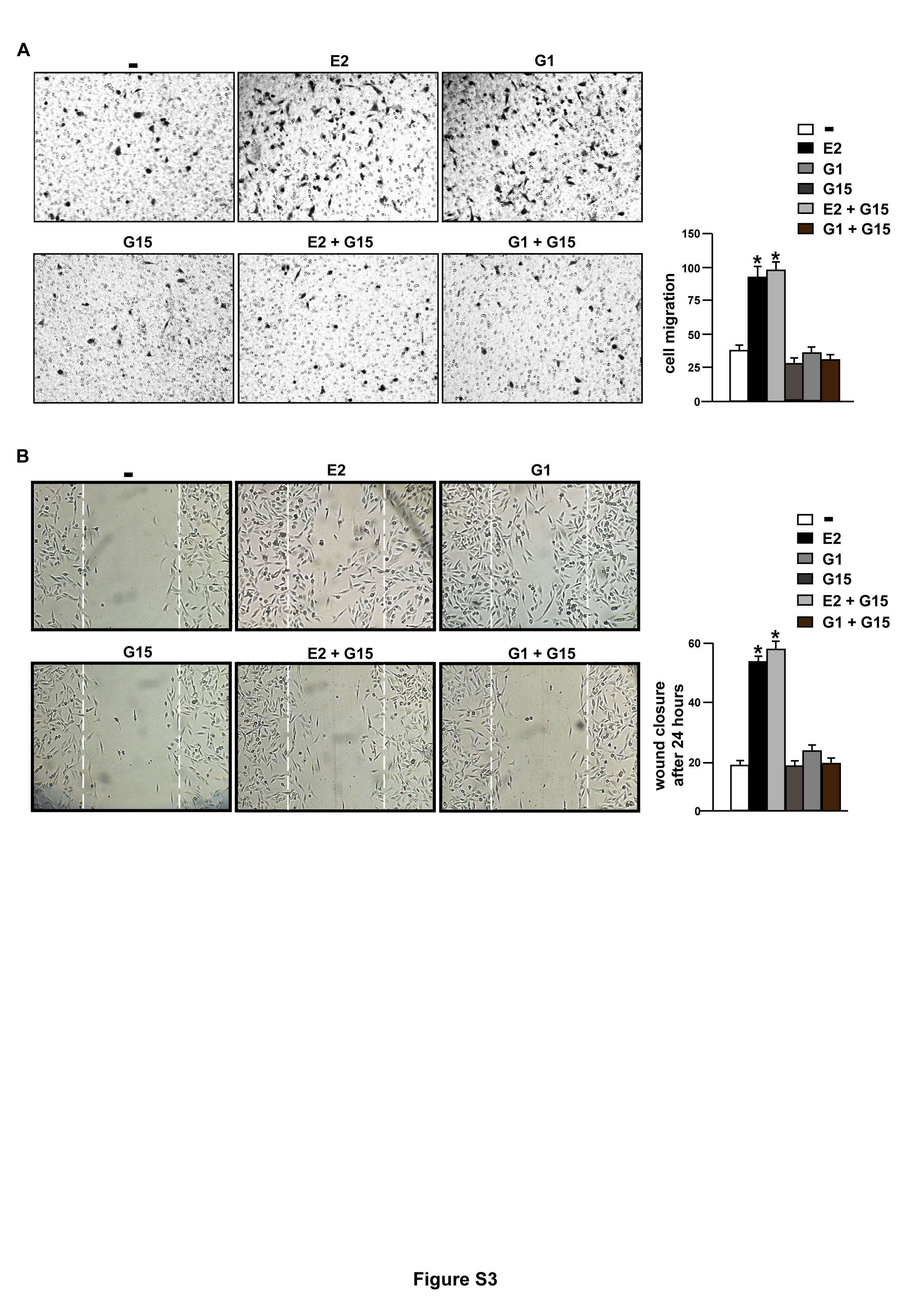

Supplement: Supplementary file 3 — Figure S3. The GPER antagonist G-15 reduces the migration of MDA-MB 231 TNBC cells induced by E2 and G1. (A) Boyden Chamber assays showing the migration of MDA-MB 231 cells treated for 4 h with 100 nM E2 and 100 nM G1 alone or in combination with 100 nM GPER antagonist G-15. The results are shown as cells migrating through the membrane at the bottom of the well upon treatments respect to vehicle (−). Results shown are representative of three independent experiments. (B) Cell migration was evaluated by wound-healing assay in MDA-MB 231 cells treated for 24 h with 100 nM E2 and 100 nM G1 alone or in combination with 100 nM GPER antagonist G-15. White dotted lines indicate the wound borders at the beginning of the assay and recorded 24 h post-scratching. Results shown are representative of three independent experiments. (*) indicates p < 0.05 [file 13046_2019_1056_MOESM3_ESM.tif]

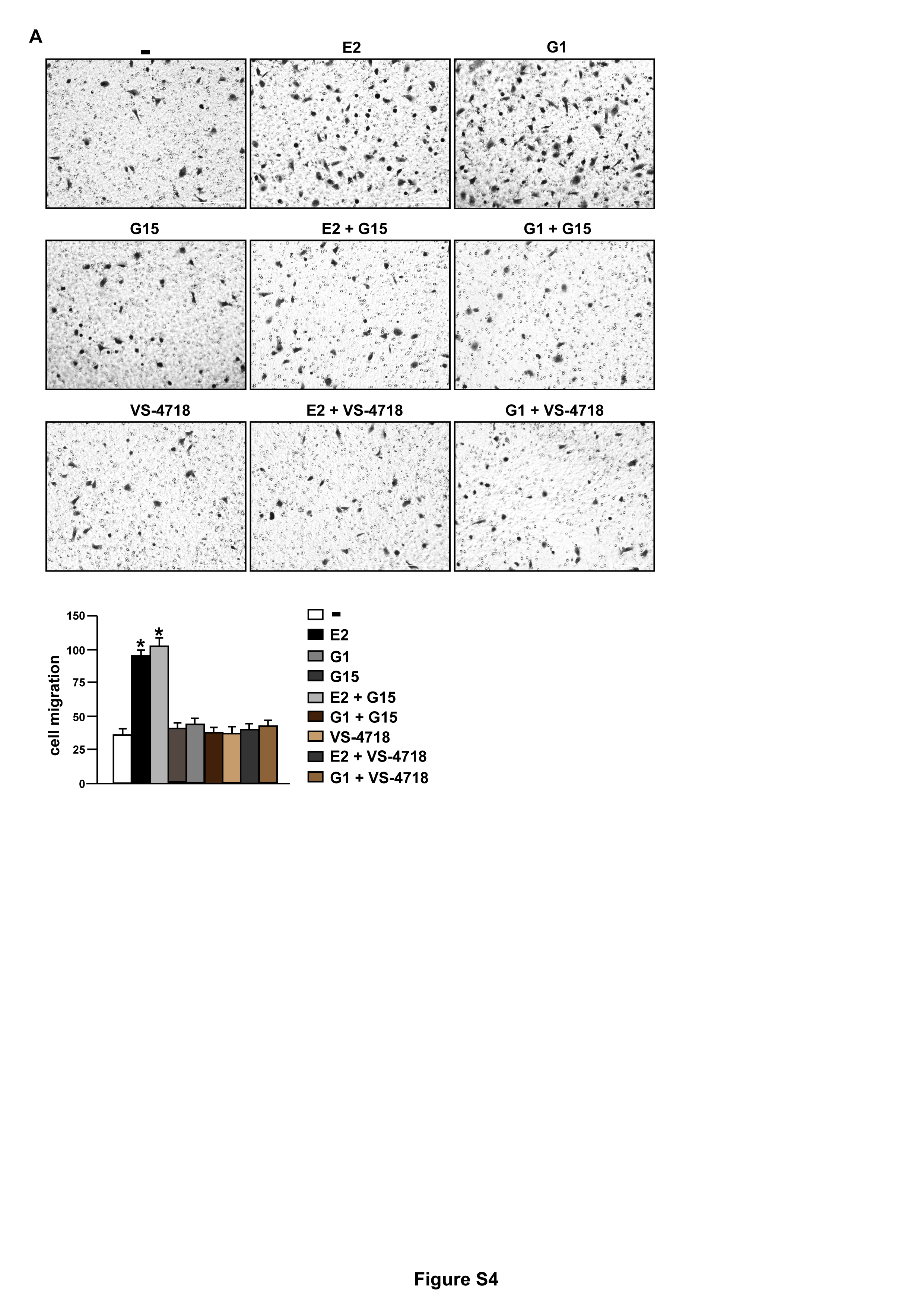

Supplement: Supplementary file 4 — Figure S4. The GPER antagonist G-15 and the FAK inhibitor VS-4718 inhibit the migration of SUM159 TNBC cells induced by E2 and G1. (A) Boyden Chamber assays showing the migration of SUM159 cells treated for 4 h with 100 nM E2 and 100 nM G1 alone or in combination with 100 nM GPER antagonist G-15 and 1 μM FAK kinase inhibitor VS-4718. The results are shown as cells migrating through the membrane at the bottom of the well upon treatments respect to vehicle (−). Results shown are representative of three independent experiments. (*) indicates p < 0.05 [file 13046_2019_1056_MOESM4_ESM.tif]
